# High expression of SOX30 is associated with favorable survival in human lung adenocarcinoma

**DOI:** 10.1038/srep13630

**Published:** 2015-09-02

**Authors:** Fei Han, Wenbin Liu, Hualiang Xiao, Yan Dong, Lei Sun, Chengyi Mao, Li Yin, Xiao Jiang, Lin Ao, Zhihong Cui, Jia Cao, Jinyi Liu

**Affiliations:** 1 Institute of Toxicology, College of Preventive Medicine, Third Military Medical University; 2 Key Laboratory of Medical Protection for Electromagnetic Radiation, Ministry of Education of China, Chongqing 400038, PR China; 3 Department of Pathology, Daping Hospital, Third Military Medical University, Chongqing, China

## Abstract

In our previous study, we had identified SOX30 as a novel tumor suppressor that acts through direct regulation of *p53* transcription in human lung cancer. Here, we sought to determine the clinical relevance of SOX30 expression in a series of surgically-resected non-small cell lung cancer (NSCLC) patients. Analysis of SOX30 expression and clinico-pathologic features reveal a significant correlation of SOX30 expression with histological type (n = 220, *P* = 0.008) and clinical stage (n = 220, *P* = 0.024). Kaplan-Meier analysis indicates an association of high SOX30 expression with better prognosis in NSCLC patients (n = 220, *P* = 0.007). Via multivariate Cox-regression analysis, SOX30 expression is revealed to be an independent prognostic factor for overall survival (OS) of NSCLC patients (n = 220, *P* = 0.014, hazard ratio (HR) = 0.816). In particular, SOX30 is a favorable and independent prognostic factor in one main subtype of NSCLC, lung adenocarcinoma (ADC) patients (n = 150, *P* = 0.000, HR = 0.405), but not in another main subtype of NSCLC, squamous cell carcinoma patients. Furthermore, high expression of SOX30 represents a favorable and independent factor for the prognosis of ADC patients at clinical stage II (*P* = 0.013), with positive lymph node (*P* = 0.003), at histological grade 2 (*P* = 0.000) or grade 3 (*P* = 0.025). In summary, SOX30 expression represents an important prognostic factor for survival time in ADC patients.

Lung cancer is the most common type of cancer and the leading cause of cancer mortality worldwide^1^,^2^. Non-small cell lung cancer (NSCLC), which mainly includes adenocarcinoma (ADC) and squamous cell carcinoma (SCC), constitutes over 70% of lung cancers^3^,^4^,^5^. The survival of NSCLC is largely dependent on clinical stages at diagnosis. At present, most cases of NSCLC are diagnosed at an advanced clinical stage due to lack of effective biomarkers. As a result the 5-year survival rate of these patients is reported to be less than 15%, despite significant improvements in diagnostics, surgery and chemotherapy^6^,^7^. To date, the best prognostic system for overall survival (OS) is still the TNM (Tumor, Node, Metastasis) staging system in NSCLC, although it is not sufficient to significantly improve the management of patients^8^. Therefore, identification of novel reliable predictive and independent prognostic factors is critical for improving therapeutic modalities and for prolonging the survival of NSCLC patients.

Sox30, a member of the Sox family of transcription factors, has been isolated from mouse, human and the Nile tilapia^9^,^10^. Sox30 is considered to be involved in gonadal development, including spermatogonial differentiation and spermatogenesis 9,10,11,12. Recently, we have demonstrated that SOX30 is a novel epigenetic silenced tumor suppressor that acts through direct regulation of *p53* in human lung cancer^13^. Although our previous studies have explored the functional role and molecular mechanism of SOX30, its clinicopathological and prognostic significance have not been clarified in lung cancer.

Here, we report on the possible roles of SOX30 as a prognostic marker for NSCLC patients. In this study, we sought to determine the clinical relevance of SOX30 expression by immunohistochemistry (IHC) on tissue microarrays (TMA) in 150 ADC and 70 SCC patients. Results indicated that SOX30 expression was significantly correlated with histological type and clinical stage of NSCLC patients. Univariate and multivariate analyses revealed that high SOX30 expression was obviously associated with better OS in NSCLC patients. Above all, SOX30 had a favorable prognostic impact on lung ADC patients, but not in SCC patients. Furthermore, SOX30 expression might represent a key prognostic factor for ADC patients at clinical stage II, with positive lymph node, at histological grade 2 or grade 3. Additionally, functional analysis revealed that SOX30 had no effect on tumor cell proliferation, cycle and apoptosis in lung SCC, which is unlike in lung ADC.

## Results

### SOX30 expression is significantly correlated with histological type and clinical stage of NSCLC patients

**Figure 1:**
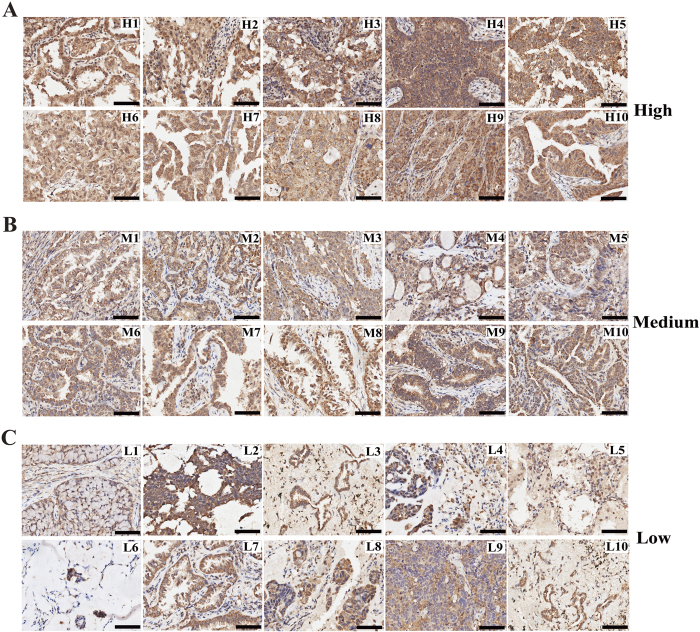
Expression level of SOX30 as seen in human NSCLC tissues. (**A**) High expression level of SOX30 as seen in representative NSCLC tissues. H, staining strong, and the expression score ≥ 9. (**B**) Medium expression level of SOX30 shown in representative NSCLC tissues. M, staining moderate, and the expression score 6< and <9. (**C**) Low expression level of SOX30 as seen in representative NSCLC tissues. L, staining weak, and the expression score ≤6. Scale bars represent 50 μm.

To determine SOX30 expression in NSCLC patients, we conducted IHC on a TMA containing 220 cancers. After IHC, we used the scoring system to consolidate the results for intensity and positive staining percentage. Based on the results, positive staining of tumor cell was quantified and classified into three groups: high (Fig. 1A), medium (Fig. 1B) and low (Fig. 1C). After investigating for possible associations between SOX30 expression and clinico-pathologic features of the patients, we found that SOX30 expression was evidently correlated to histological type (n = 220, P = 0.008) and clinical stage (n = 220, P = 0.024) of NSCLC patients (Table 1). The incidence of SOX30 over-expression was 31.33% (47/150) in ADC, 14.29% (10/70) in SCC, 29.35% (27/92) in clinical stage I, 26.19% (11/42) in clinical stage II and 13.64% (9/66) in clinical stage III+IV of NSCLC patients, respectively (Table 1). However, SOX30 expression was not correlated to age (*P *= 0.535), gender (*P *= 0.052), histological grade (*P *= 0.516), tumor size (*P *= 0.086) and lymph node status (*P *= 0.533) (Table 1). In addition, we also found that SOX30 expression was correlated to clinical stage (n = 150, P = 0.036) of ADC patients, and the incidence of SOX30 over-expression was 35.29% (24/68) in clinical stage I, 29.17% (7/24) in clinical stage II and 15.79% (6/38) in clinical stage III+IV of ADC patients, respectively (Table S1).

### Increased SOX30 expression is obviously associated with better OS of NSCLC patients

**Figure 2:**
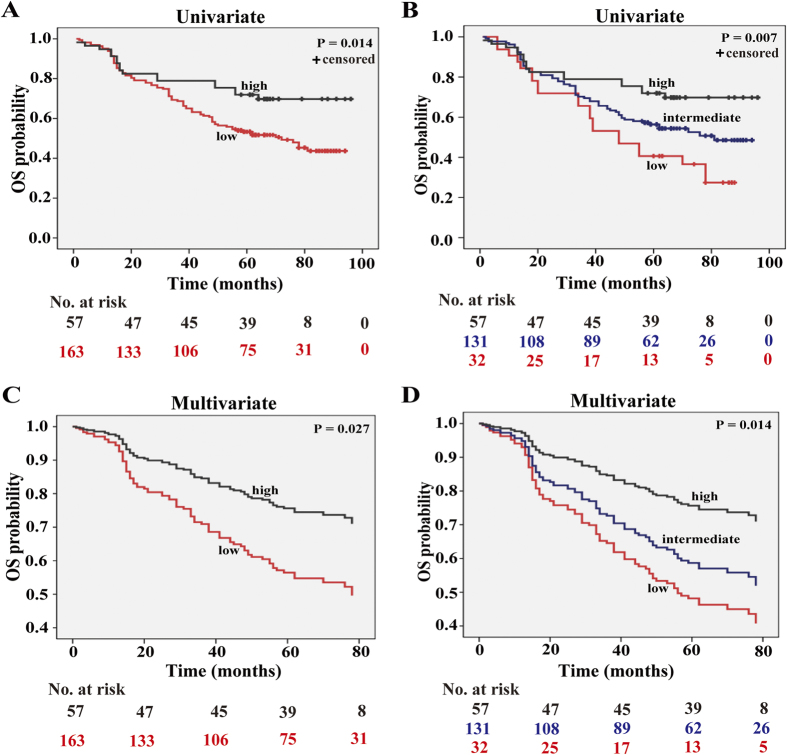
High expression of SOX30 is correlated with long survival time in NSCLC patients. (**A**) Survival analysis of SOX30 expression in 220 NSCLC patients. Patients were split into two groups by Kaplan-Meier survival curve. Tissue array analysis was performed for 220 cases of NSCLC patients with survival information. Compared to patients with high SOX30 expression, patients with low SOX30 expression had an inferior OS rate; Low, staining weak and moderate; High, staining strong. (**B**) Kaplan-Meir survival analysis of SOX30 expression in 220 NSCLC patients split into three groups. Longer OS was observed in the higher SOX30 group as compared to the lower SOX30 group. Low, staining weak; Medium, staining moderate; High, staining strong. (**C**) Survival analysis of SOX30 expression in 220 NSCLC patients split into two groups by multivariate Cox regression. SOX30 expression was determined to be an independent prognostic factor. (**D**) Cox regression survival analysis of SOX30 expression in 220 NSCLC patients split into three groups. Longer OS was determined for the higher SOX30 group as compared to the lower SOX30 group. SOX30 protein expression was an independent prognostic factor of survival.

We then combined low and intermediate scores to obtain two groups of SOX30 expression: low and high. SOX30 expression groups were analyzed with respect to patient survival data. Survival analysis using Kaplan-Meier and log rank test revealed poorer OS in NSCLC patients characterized with low SOX30 expression as compared to patients with high SOX30 expression (*P* = 0.014) (Fig. 2A). This result was confirmed by the survival analysis of three directly obtained groups: low, intermediate and high (*P* = 0.007) (Fig. 2B). To correct for bias caused by univariate analysis, SOX30 expression as well as other parameters were examined in a multivariate Cox-regression analysis (after adjustment for age, clinical stage, gender, histological grade, tumor size and lymph node). In addition to age (hazard ratio (HR) = 1.057, *P* = 0.000) and clinical stage (HR = 1.858, *P* = 0.000), SOX30 expression was found to be an independent prognostic factor (HR = 0.816, *P* = 0.027 for two groups/HR = 0.736, *P* = 0.014 for three groups) for the OS of NSCLC patients (Fig. 2C,D, Table S2).

To further confirm our results, we then analyzed the clinical significance of SOX30 mRNA expression in human lung cancers with Kaplan-Meier Plotter (http://kmplot.com/analysis/index.php?p = background), an online tool to evaluate the correlation of SOX30 expression (207678_s_at) with lung cancer prognosis in over 1400 clinical patients. We found that higher mRNA expression of SOX30 was linked to markedly longer OS of lung cancers (Figure S1A, HR = 0.76, p = 0.00065). In a multivariable analysis adjusted for histology, grade, stage, AJCC stage T, AJCC stage N, gender, age, smoking history, the patients with higher mRNA expression of SOX30 also had a better prognosis (Figure S1B, HR = 0.4, p = 0.0024). In consideration of the gene expression data of the KMplotter database from different microarray analysis platforms, we analyzed the clinical significance of SOX30 mRNA expression with Kaplan-Meier Plotter in human lung cancers from three selected cohorts: the TCGA dataset (Figure S1C, HR = 0.31, p = 0.0075), GSE19188 dataset (Figure S1D, HR = 0.4, p = 0.0023) and GSE4573 dataset (Figure S1E, HR = 0.41, p = 0.0028) respectively. We also found that higher mRNA expression of SOX30 was linked to markedly longer OS of lung cancers as figure S1C-E.

### High expression of SOX30 suggests favorable survival outcomes in ADC patients

**Figure 3:**
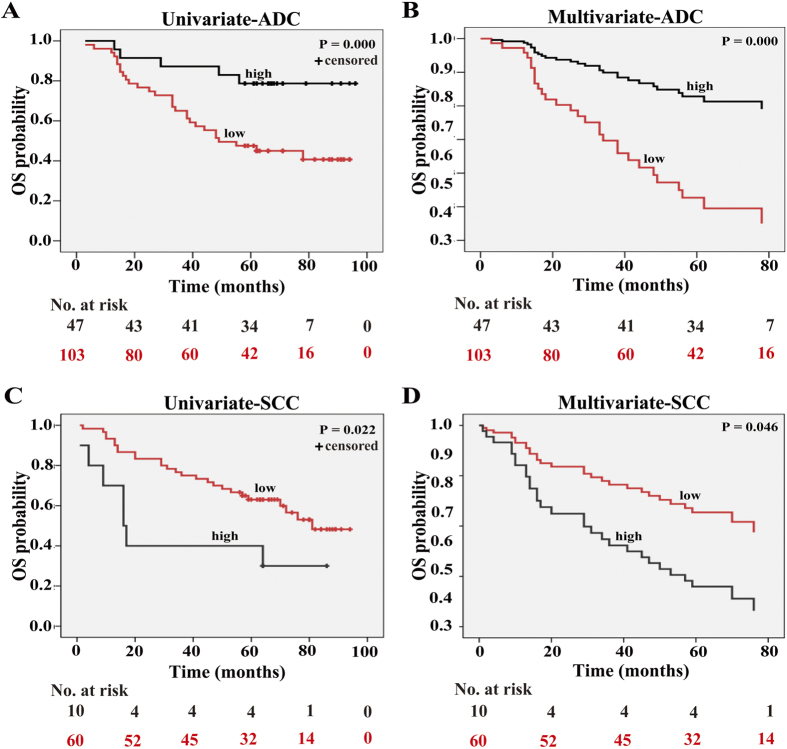
SOX30 level has a positive prognostic significance in ADC patients but a negative prognostic significance in SCC patients. (**A**) Survival analysis of SOX30 expression in 150 ADC patients by Kaplan-Meier method. Patients were split into two groups. Compared to patients with low SOX30 expression, patients with high SOX30 expression had a good OS rate; Low, staining weak and moderate; High, staining strong. (**B**) Survival analysis of SOX30 expression in 150 ADC patients by multivariate Cox regression. Patients were split into two groups. SOX30 expression was determined to be an independent prognostic factor. (**C**) Kaplan-Meir survival analysis of SOX30 expression in 70 SCC patients. Long OS was observed in the low SOX30 group as compared to the high SOX30 group. Low, staining weak and moderate; High, staining strong. (D) Cox regression survival analysis of SOX30 expression in 70 SCC patients. SOX30 expression was an independent prognostic factor of survival.

To investigate the correlations between SOX30 expression and survival of ADC and SCC patients respectively, the prognostic significance of SOX30 was analyzed. In ADC patients, Kaplan Meier analysis indicated that patients with high levels of SOX30 expression had significantly prolonged OS compared to those with low levels of SOX30 expression (p = 0.000, Fig. 3A). To avoid the influence caused by univariate analysis, the multivariate Cox regression analysis was performed, and SOX30 expression was identified as an independent prognostic factor (HR = 0.405, p = 0.000), in addition to age (HR = 1.056, p = 0.001) and clinical stage (HR=1.962, p = 0.000) in ADC patients (Fig. 3B and Table 2). In SCC patients, univariate analysis demonstrated that patients with high SOX30 expression had poorer OS than those with low SOX30 expression (p = 0.022, Fig. 3C). Moreover, multivariate Cox regression analysis showed that SOX30 expression was also an independent prognostic factor (HR = 1.283, p = 0.046), in addition to age (HR = 1.067, p = 0.024) in SCC patients (Fig. 3D, Table S3). These findings revealed that high SOX30 expression was a favorable and independent prognostic factor for ADC patients, but not for SCC patients.

To further validate the result above, we also analyzed the clinical significance of SOX30 mRNA expression with Kaplan-Meier Plotter in lung ADC and SCC respectively. The data from analyses of public dataset is consistent with our study in lung ADC (Figure S1F, HR = 0.60, p = 0.00067), but is not in lung SCC. Unfortunately, in the three selected cohorts, we failed to analyze the clinical significance of SOX30 mRNA expression in lung ADC and SCC respectively, because of small case number.

### High expression of SOX30 indicates better survival of clinical stage II or lymph node-positive ADC patients

**Figure 4:**
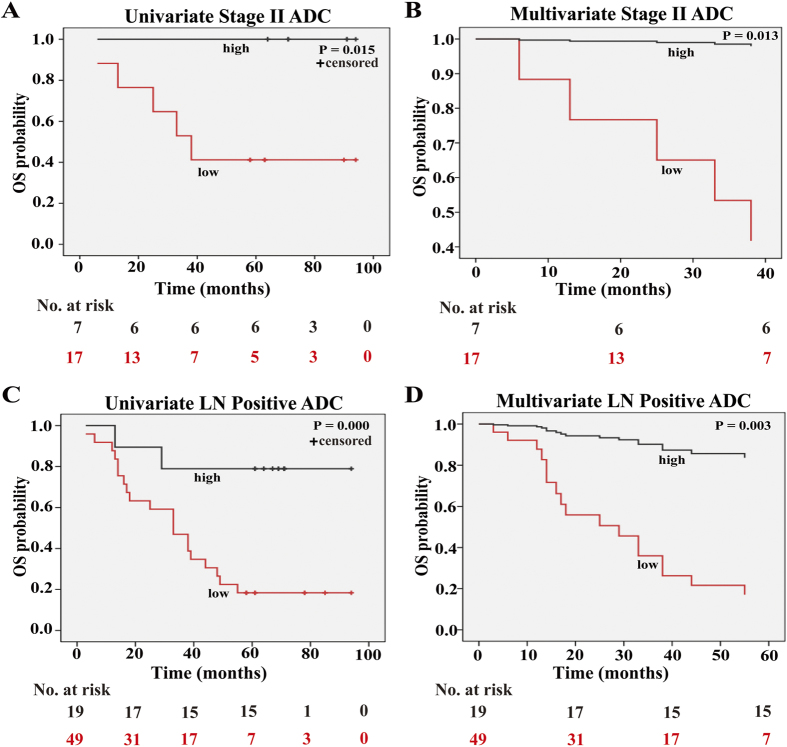
High expression of SOX30 is correlated with better OS in ADC patients at clinical stage II or with lymph node-positive. (**A**) Kaplan-Meir survival analysis of SOX30 expression in 24 clinical stage II ADC patients. The patients with high SOX30 expression had a good OS rate; Low, staining weak and moderate; High, staining strong. (**B**) Cox regression survival analysis of SOX30 expression in 24 clinical stage II ADC patients. SOX30 expression was an independent prognostic factor. (**C**) Kaplan-Meir survival analysis of SOX30 in 68 ADC patients with lymph node-positive. The high SOX30 expression group had a good OS rate; Low, staining weak and moderate; High, staining strong. LN Positive, lymph node-positive. (**D**) Cox regression survival analysis of SOX30 expression in 68 ADC patients with lymph node-positive. SOX30 expression was an independent prognostic factor in these patients.

To determine the importance of clinical stage and lymph node status on the correlation between SOX30 expression and OS of ADC patients, we stratified patients by SOX30 expression and clinical stage or lymph node status, followed by analysis of survival data. The results revealed that SOX30 expression was not statistically associated with OS of ADC patients at clinical stage I (*P* = 0.215), stage III (P = 0.156), stage III+IV (*P* = 0.223) or with negative lymph node (*P* = 0.224). However, high SOX30 expression was significantly correlated with better OS of stage II (univariate analysis: *P* = 0.015/multivariate analysis: *P* = 0.013) or lymph node-positive (*P* = 0.000/*P* = 0.003) ADC patients (Fig. 4A–D). These findings suggested that high SOX30 expression was a favorable and independent prognostic factor for stage II or lymph node-positive ADC patients.

### High expression of SOX30 predicts better outcome of histological grade 2 or grade 3 ADC patients

**Figure 5:**
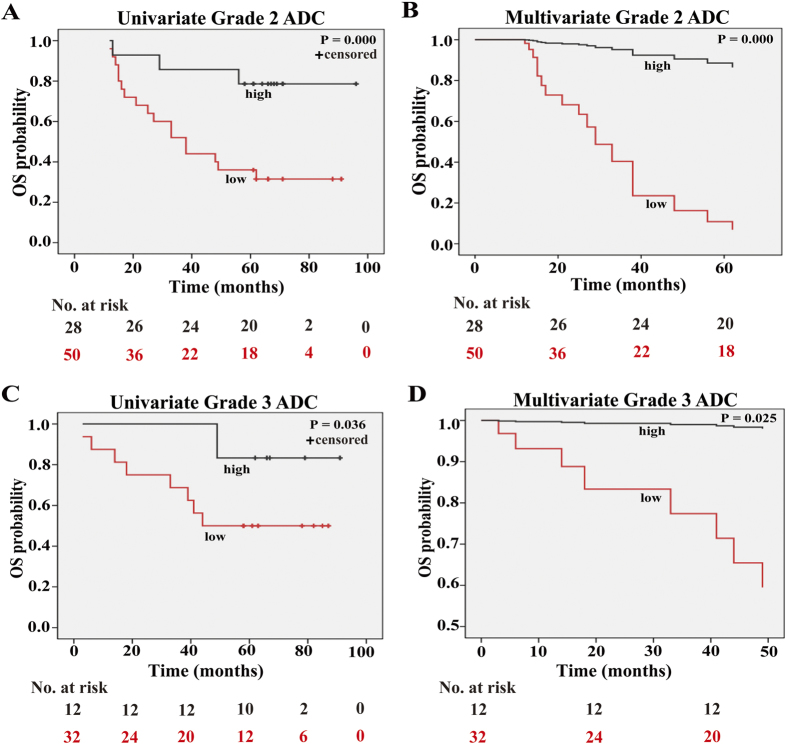
High expression of SOX30 is associated with better OS in ADC patients at histological grade 2 or 3. (**A**) Kaplan-Meir of SOX30 expression in 78 histological grade 2 ADC patients. The group with high SOX30 expression had a good OS rate; Low, staining weak and moderate; High, staining strong. (**B**) Cox regression survival analysis of SOX30 expression in 78 histological grade 2 ADC patients. The expression of SOX30 was an independent prognostic factor. (**C**) Kaplan-Meir of SOX30 expression in 44 histological grade 3 ADC patients. The group with high SOX30 expression had a good OS rate; Low, staining weak and moderate; High, staining strong. (**D**) Cox regression survival analysis of SOX30 expression in 44 histological grade 3 ADC patients. The expression of SOX30 was an independent prognostic factor.

Next, we sought to determine the impact of SOX30 expression on patient survival while considering histological grade. After stratifying patients based on SOX30 expression, we analyzed patient survival data in correlation to histological grade. High SOX30 expression was evidently correlated to better survival in histological grade 2 (univariate analysis: *P* = 0.000/multivariate analysis: *P* = 0.000) or grade 3 (*P* = 0.036/*P* = 0.025) ADC patients (Fig. 5A–D). However, SOX30 expression was not correlated with survival in histological grade 1 (*P* = 0.755) ADC patients. These results indicated that high SOX30 expression was a favorable and independent prognostic factor for histological grade 2 or grade 3 ADC patients.

### SOX30 induces cancer cell apoptosis with inhibiting proliferation in lung ADC, but not in lung SCC

**Figure 6:**
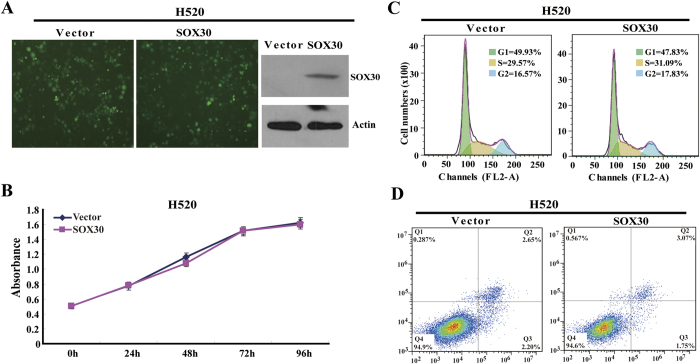
Analyses of cell proliferation, cell cycle and apoptosis associated with SOX30 over-expression in NCI-H520 cells. (**A**) Transfectants of SOX30 and vector control in NCI-H520 cells were identified by WB. Abundant SOX30 was detected after SOX30 transfection but not after the vector control transfection. Error bars indicate s.d. (n = 3). (**B**) MTS assays were used to examine the effect of SOX30 on cell proliferation in NCI-H520 cells. Cell viability was evaluated in triplicate by CellTiter 96 AQueous One Solution Cell Proliferation Assay (Promega). (**C**) Cell-cycle profiles were determined by flow cytometry. (**D**) Cell apoptosis was performed by flow cytometry assay with Annexin V-APC/7-amino-actinomycin D double staining in NCI-H520 cells. Three independent experiments were performed.

Our previous studies have showed that SOX30 functions as a tumor suppressor mainly through promoting tumor cell apoptosis with inhibiting proliferation in lung ADC^13^. To explore the potential role of SOX30 in lung SCC, we generated gain-of-function cell models by transfecting a SOX30-expressing construct into the human SCC NCI-H520 (H520) cell line (Fig. 6A). We then examined the effect of SOX30 over-expression on cell proliferation and viability. Five-day growth curve analysis showed that over-expression of SOX30 did not affect proliferation of H520 cells (Fig. 6B). To further confirm the result, we measured the percentage of cell cycle and conducted Annexin V-APC/7-amino-actinomycin D double staining followed by flow cytometry analysis. The results showed that SOX30 also did not affect H520 cell cycle and apoptosis (Fig. 6C,D). Taking our previous study into account^13^, the functional data reveal that SOX30 is a tumor suppressor gene in lung ADC, but has no effect on tumor cell proliferation, cycle and apoptosis in lung SCC.

## Discussion

In the present study, we investigated for the first time the clinical relevance of SOX30 expression in NSCLC patients. From our data, we find that SOX30 expression is obviously correlated with histological type and clinical stage of NSCLC patients. Kaplan-Meier analysis reveals that the patients with increased expression levels of SOX30 have correspondingly prolonged OS compared to patients characterized by low SOX30 expression. Multivariate Cox-regression analysis suggests that SOX30 is an independent prognostic factor for OS in NSCLC patients. In particular, SOX30 is a favorable and independent prognostic factor in lung ADC patients, but not in lung SCC patients. Furthermore, SOX30 expression represents a favorable and independent prognostic factor for ADC patients at clinical stage II (please see Table S4 (ADC-150-Sox30) of Supplementary data (excel) for the detail of the staging system used), with positive lymph nodes, at histological grade 2 or grade 3. These data suggest that SOX30 protein expression is an independent predictor of favorable prognosis in ADC patients.

Accumulated evidence have indicated that different histologic subtypes of NSCLC showed different molecular events during tumorigenesis^14^,^15^, suggesting the importance of characterizing molecular abnormalities in those tumor types separately. In our study, SOX30 expression is remarkably correlated with histological type of NSCLC patients, suggesting a different clinicopathological significance of SOX30 in ADC and SCC patients. Our further studies by univariate and multivariate analyses demonstrates that high SOX30 expression significantly correlates with better outcome in ADC patients, but not in SCC patients. However, this result was not well validated from analyses of the public dataset. There maybe two reasons: 1) the gene expression data of the KMplotter database are from different microarray analysis platforms; 2) the SOX30 expression data of the dataset are mRNA levels, while the SOX30 expression in our data is protein level, and in many cases the changes of gene expression in mRNA level do not always reflect the changes in protein level.

Previous studies have shown that histological type may be a major cause of heterogeneity of NSCLC patients in different studies^16^. Recently, specific genetic abnormalities have been identified in NSCLC, which shows distinct features according to the histologic types. In general, activating mutations of EGFR, ALK and KRAS are mainly identified in ADC patients, whereas p53 mutation appears to be more frequent in SCC patients^17^. These dysregulations influence apoptosis signaling pathways in NSCLC patients. In particular, our previous study revealed that SOX30 acts as a tumor suppressor by promoting cancer cell apoptosis through directly regulation of p53 transcription in ADC patients^13^, suggesting a positive prognostic impact of SOX30. However, SOX30 may not function as a tumor suppressor if it only promotes cell apoptosis through the direct regulation of p53 transcription in SCC patients with p53 mutation. Therefore, an explanation for the different prognostic role of SOX30 may be that different functional roles of SOX30 cause different prognostic effects in ADCs and SCCs. To explore the potential role of SOX30 in SCCs, we generated gain-of-function cell models, and then examined the effect of SOX30 over-expression on cell proliferation, cell cycle and apoptosis. The results show that SOX30 do not affect NCI-H520 cell proliferation, cell cycle and apoptosis (Fig. 6A–D). From our previous and present studies, SOX30 is a tumor suppressor in ADC, but has no effect on tumor cell proliferation, cell cycle and apoptosis in SCC. This is probably the reason for the opposite results of SOX30 expression in survival outcome between ADC and SCC.

As the efficacy of treatment options for cancer varies in different patient subgroups, the finding of useful predictive and prognostic markers are necessary for improving patient survival^18^,^19^. At present, many factors, such as age, tumor size and lymph node status, have been used to predict the outcome of cancer patients^20^,^21^. However, these factors are unable to determine the cancer patient’s individual risk. Thus, identification of new clinically-relevant prognostic markers are still of great importance. In the present study, we describe the association between SOX30 expression and survival with regards to clinical stage, lymph node status and histological grade in ADC patients. Our data suggest that high SOX30 expression is correlated with a favorable prognosis for ADC patients at clinical stage II, with positive lymph nodes, at histological grade 2 or grade 3. From these results, we proposed SOX30 expression as an independent predictor of favorable prognosis for clinical stage II, lymph node-positive, histological grade 2 or grade 3 lung ADC patients.

In our study, the five-year overall survival rate is over 40% for NSCLC patients^22^,^23^,^24^,^25^, as most cases of patients who require surgery are diagnosed at a early clinical stage (I and II). However, we report a different follow-up period using survival curves as in Fig. 3. After a careful check, it was found that this difference could be attributed to missing information in certain cases, and to multivariate analyses that were negatively impacted by the lack of complete patient information. From our data, the significant better OS for high expression of SOX30 was found only in the subset of stage II and not in stage I, which seemed to be confusing. Considering that the number of patients with stage II is rather small, it also needs to be validated in a larger series. Previous studies have indicated that the value of events per variable should equal 10^26^. In the present study, several factors were characterized with a lower event per variable value. Therefore, we suggest revalidation of some of the results in a study with a larger sample size. Additionally, although our data represent SOX30 expression as an independent predictor of favorable prognosis in ADC patients, the predictive role of it for treatment response should be further explored in upcoming clinical trials.

In conclusion, SOX30 is significantly associated with histologic type and clinical stage in NSCLC patients, and heightened SOX30 represents an independent prognostic factor for increased survival time of ADC patients or the ones who are at clinical stage II, with positive lymph nodes, at histological grade 2 or grade 3.

## Materials and Methods

### Patient samples

A total of 220 primary NSCLC patients including 150 ADCs and 70 SCCs, who had undergone surgical resection with curative intent between 2004 and 2007, were obtained from the Southwest Hospital in Chongqing, China. The clinico-pathologic information was retrieved from the patients’ electronic medical records, which included age, gender, lymph node (negative or positive), tumor size, histological grade and clinical stage defined according to AJCC (American Joint Committee on Cancer) 7th edition and the new TNM classification^27^, and follow-up information (5-8 years) for OS rates. This study was approved by the ethics committee of the Southwest Hospital Affiliated to Third Military Medical University, and all experiments were carried out in accordance with approved guidelines of Third Military Medical University. Informed consent was signed by all of the recruited patients.

### Tissue microarray (TMA) generation

All samples from NSCLC patients were reviewed histologically by hematoxylin and eosin staining. To construct the TMA slides, two cores were taken from each representative tumor and adjacent noncancerous tissue (within a distance of 20 mm). The non-cancerous adjacent tissues were compared with normal tissue, stained with hematoxylin-eosin then reviewed histologically by at least two pathologists. Duplicate cylinders from intratumoral and peritumoral areas were obtained. Finally, the TMAs were constructed (in collaboration with Shanghai Biochip Company Ltd, Shanghai, China)^28^.

### Immunohistochemical analysis

IHC was performed using SOX30 antibody (1:100; Santa Cruz Biotechnology) as described previously^12^. Tumor cell staining was evaluated and considered positive when immunoreactivity was greater than or equal to 10%. Based on IHC, positive staining was quantified and classified into 5 categories: <10% positive cells for 0 (score); 10% to 25% for 1; 26% to 50% for 2; 51% to 75% for 3 and ≥76% for 4. Staining intensity was graded as negative (scored as 0), weak (1), moderate (2) or strong (3). All core biopsies were independently reviewed by two pathologists, and expression levels were defined by the sum of the grades for the percentage of positive staining and intensity.

### Construction of SOX30 expression vector, cell transfection, MTS and Flow cytometry assays

Construction of SOX30 expression vector, cell transfection, MTS assay and Flow cytometry assay were performed as previously described^13^.

### Statistical Analysis

Statistical analyses were performed using the SPSS 16.0 software (SPSS, Inc., Chicago, IL). The difference in categorical variables was analyzed by Chi-square test and Linear-by-Linear Association (2-sided). OS was calculated according to Kaplan-Meier method and evaluated by log-rank test. Cox regression was used for multivariate analysis of prognostic predictors. SOX30 expression was categorized as high or low using the median score^29^. A p value of less than 0.05 was taken as statistically significant.

## Additional Information

**How to cite this article**: Han, F. *et al.* High expression of SOX30 is associated with favorable survival in human lung adenocarcinoma. *Sci. Rep.* **5**, 13630; doi: 10.1038/srep13630 (2015).

## Supplementary Material

Supplementary Information

Supplementary Dataset

## Figures and Tables

**Table 1:** Correlations of SOX30 expression with clinico-pathologic features in human NSCLC patients (n = 220).

**SOX30 Expression**					
**Clinical Feature**	**Total**	**High (n = 57)**	**Medium (n = 131)**	**Low (n = 32)**	**P value**
Age (years)					
<60	95	27	54	14	0.535
≥60	125	30	77	18	
Histological type					
ADC	150	47	85	18	**0.008**
SCC	70	10	46	14	
Clinical stage					
I	92	27	51	14	
II	42	11	29	2	**0.024**
III	59	7	39	13	
IV	7	2	4	1	
Gender					
Male	144	31	90	23	0.052
Female	76	26	41	9	
Histological grade					
1	37	7	22	8	0.516
2	118	33	72	13	
3	62	17	34	11	
Tumor size					
≤3 cm	86	28	45	13	0.086
>3 cm	132	29	84	19	
Lymph node status					
Negative	117	33	69	15	0.533
Positive	96	23	57	16	

**Table 2:** Multivariate analysis of different prognostic factors in human lung ADC patients (n = 150).

**Expression level**	**Variable**	**HR**	**95% CI**	**P value**
The protein level expression of SOX30	Age	1.056	1.024–1.090	**0.001**
	Gender	0.796	0.410–1.546	0.500
	Clinical stage	1.962	1.393–2.765	**0.000**
	Histological grade	0.968	0.599–1.565	0.895
	Tumor size	0.860	0.698–1.060	0.157
	Lymph node status	1.036	0.955–1.123	0.397
	SOX30 expression	0.405	0.112–0.561	**0.000**

Abbreviations: HR, hazard ratio; CI, confidence interval.

## References

[b1] Siegel R., Naishadh D. & Jemal A. Cancer statistics. CA. Cancer. J. Clin. 63, 11–30 (2013). 23335087 10.3322/caac.21166

[b2] Ferlay J. *et al.* GLOBOCAN 2012 v1.0, Cancer Incidence and Mortality Worldwide: IARC CancerBase No. 11 [Internet]. Lyon, France: International Agency for Research on Cancer., 2013. Available from: http://globocan.iarc.fr, accessed on 23/12/2014.

[b3] Ridge C. A., McErlean A. M. & Ginsberg M. S. Epidemiology of lung cancer. Semin. Interv. Radiol . 30, 93–98 (2013). 10.1055/s-0033-1342949 PMC3709917 24436524

[b4] Pao W. & Chmielecki J. Rational, biologically based treatment of EGFR-mutant non-small-cell lung cancer. Nat. Rev. Cancer. 10, 760–774 (2010). 20966921 10.1038/nrc2947 PMC3072803

[b5] Herbst R. S., Heymach J. V. & Lippman S. M. Lung cancer. N. Engl. J. Med. 359, 1367–1380 (2008). 18815398 10.1056/NEJMra0802714 PMC10662965

[b6] Molina J. R., Yang P., Cassivi S. D., Schild S. E. & Adjei A. A. Non-small cell lung cancer: epidemiology, risk factors, treatment, and survivorship. Mayo. Clin. Proc. 83, 584–594 (2008). 18452692 10.4065/83.5.584 PMC2718421

[b7] Jemal A., Siegel R., Xu J. & Ward E. Cancer statistics, 2010. CA. Cancer. J. Clin. 60, 277–300 (2010). 20610543 10.3322/caac.20073

[b8] Parkin D. M., Bray F., Ferlay J. & Pisani P. Global cancer statistics, 2002. CA. Cancer. J. Clin. 55, 74–108 (2005). 15761078 10.3322/canjclin.55.2.74

[b9] Osaki E. *et al.* Identification of a novel Sry-related gene and its germ cell-specific expression. Nucleic. Acids. Res. 27, 2503–2510 (1999). 10359848 10.1093/nar/27.12.2503 PMC148454

[b10] Han F. *et al.* Characterization, phylogeny, alternative splicing and expression of Sox30 gene. BMC Molecular Biology. 11, 98 (2010). 21143990 10.1186/1471-2199-11-98 PMC3004900

[b11] Ballow D., Meistrich M. L., Matzuk M. & Rajkovic A. Sohlh1 is essential for spermatogonial differentiation. Dev. Biol. 294, 161–167 (2006). 16564520 10.1016/j.ydbio.2006.02.027

[b12] Han F. *et al.* Epigenetic regulation of Sox30 is associated with testis development in mice. PLoS One. 9, e97203 (2014). 24810894 10.1371/journal.pone.0097203 PMC4014610

[b13] Han F. *et al.* SOX30, a novel epigenetic silenced tumor suppressor, promotes tumor cell apoptosis by transcriptional activating p53 in lung cancer. *Oncogene*. Epub ahead of print (2014). 10.1038/onc.2014.370 PMC4541146 25435374

[b14] McDoniels-Silvers A. L., Stoner G. D., Lubet R. A. & You M. Differential expression of critical cellular genes in human lung adenocarcinomas and squamous cell carcinomas in comparison to normal lung tissues. Neoplasia. 4, 141–150 (2002). 11896569 10.1038/sj.neo.7900217 PMC1550320

[b15] Pao W. & Girard N. New driver mutations in non-small-cell lung cancer. Lancet Oncol. 12, 175–180 (2011). 21277552 10.1016/S1470-2045(10)70087-5

[b16] Huang L. N. *et al.* Expression of survivin and patients survival in non-small cell lung cancer: a meta-analysis of the published studies. Mol. Biol. Rep. 40, 917–924 (2013). 23065255 10.1007/s11033-012-2132-8

[b17] Perez-Moreno P., Brambilla E., Thomas R. & Soria J. C. Squamous cell carcinoma of the lung: molecular subtypes and therapeutic opportunities. Clin. Cancer. Res. 18, 2443–2451 (2012). 22407829 10.1158/1078-0432.CCR-11-2370

[b18] Cho W. C. Great potential of miRNAs as predictive and prognostic markers for cancer. Expert. Rev. Mol. Diagn. 12, 315–318 (2012). 22616694 10.1586/erm.12.21

[b19] Peng L., Song Z. G. & Jiao S. C. Efficacy analysis of tyrosine kinase inhibitors on rare non-small cell lung cancer patients harboring complex EGFR mutations. Sci Rep . 4, 6104 (2014). 25130612 10.1038/srep06104 PMC4135336

[b20] Elledge R. M., McGuire W. L. & Osborne C. K. Prognostic factors in breast cancer. Semin. Oncol. 19, 244–253 (1992). 1351692

[b21] Hayes D. F. Tumor markers for breast cancer. Ann. Oncol. 4, 807–819 (1993). 8117599 10.1093/oxfordjournals.annonc.a058385

[b22] Qu X., Zhang T., Ma H., Sui P. & Du J. Lower mean corpuscular hemoglobin concentration is associated with unfavorable prognosis of resected lung cancer. Future. Oncol . 10, 2149–2159 (2014). 25471030 10.2217/fon.14.121

[b23] Fukumoto K. *et al.* The ABO blood group is an independent prognostic factor in patients with resected non-small cell lung cancer. J. Epidemiol. Epub ahead of print (2014). 10.2188/jea.JE20140102 PMC4310871 25483106

[b24] Potprommanee L. *et al.* GM2-Activator Protein: A New Biomarker for Lung Cancer. J. Thorac. Oncol. Epub ahead of print (2014). 10.1097/JTO.0000000000000357 25490003

[b25] Lee S. H. *et al.* Reactive oxygen species modulator 1 (Romo1) overexpression is an independent predictor of poor survival in NSCLC patients who undergo surgical resection. Lung Cancer. 87, 45–52 (2015). 25468147 10.1016/j.lungcan.2014.11.004

[b26] Peduzzi P., Concato J., Feinstein A. R. & Holford T. R. Importance of events per independent variable in proportional hazards regression analysis. II. Accuracy and precision of regression estimates. J. Clin. Epidemiol. 48, 1503–1510 (1995). 8543964 10.1016/0895-4356(95)00048-8

[b27] Goldstraw P. *et al.* The IASLC Lung Cancer Staging Project: proposals for the revision of the TNM stage groupings in the forthcoming (seventh) edition of the TNM Classification of malignant tumours. J. Thorac. Oncol. 2, 706–714 (2007). 17762336 10.1097/JTO.0b013e31812f3c1a

[b28] Gao Q. *et al.* Intratumoral balance of regulatory and cytotoxic T cells is associated with prognosis of hepatocellular carcinoma after resection. J. Clin. Oncol. 25, 2586–2593 (2007). 17577038 10.1200/JCO.2006.09.4565

[b29] Huang G. S. *et al.* Insulin-like growth factor 2 expression modulates Taxol resistance and is a candidate biomarker for reduced disease-free survival in ovarian cancer. Clin. Cancer. Res. 16, 2999–3010 (2010). 20404007 10.1158/1078-0432.CCR-09-3233 PMC2887721

