# Supplementary material for: High expression of SOX30 is associated with favorable survival in human lung adenocarcinoma
Source: Sci Rep. 2015 Sep 2;5:13630. doi: 10.1038/srep13630 (PMC4557060; doi:10.1038/srep13630)
Supplement: Supplementary Information [file srep13630-s1.pdf]

High expression of SOX30 is associated with favorable survival in human lung adenocarcinoma

Fei Han[1,2], Wenbin Liu[1,2], Hualiang Xiao[3], Yan Dong[1,2], Lei Sun[1,2], Chengyi Mao[3], Li Yin[1,2], Xiao Jiang[1,2], Lin Ao[1,2], Zhihong Cui[1,2], Jia Cao [1,2,*], Jinyi Liu [1,2,*]

Supplementary Tables

Table S1 Correlations of SOX30 expression with clinicopathologic features in human ADC patients (n=150)

Clinical Feature	Total	SOX30 Expression		P value
		High (n=47)	Low (n=103)	
Age (years)				
< 60	76	25	51	0.860
≥60	72	22	50	
Clinical stage				
I	68	24	44	**0.036**
II	24	7	17	
III+IV	38	6	32	
Gender				
Male	78	23	55	0.725
Female	72	24	48	
Histological grade				
1	26	7	19	0.521
2	78	28	50	
3	44	12	32	
Tumor size				
≤ 3cm	70	26	44	0.163
> 3cm	80	21	59	
Lymph node status				
Negative	80	28	52	0.381
Positive	68	19	49	

Table S2 Multivariate analysis of different prognostic factors in human NSCLC patients (n=220)

Expression level	Variable	HR	95% CI	P value
The protein level expression of SOX30	Age	1.057	1.031-1.084	**0.000**
	Gender	1.091	0.621-1.915	0.763
	Clinical stage	1.858	1.376-2.507	**0.000**
	Histological type	0.732	0.430-1.246	0.251
	Histological grade	0.944	0.643-1.385	0.768
	Tumor size	1.003	0.872-1.153	0.972
	Lymph node status	1.065	0.987-1.150	0.104
	SOX30 expression	0.816	0.713-0.980	**0.027**

Abbreviations: HR, hazard ratio; CI, confidence interval

Table S3 Multivariate analysis of different prognostic factors in human lung SCC patients (n=70)

Expression level	Variable	HR	95% CI	P value
The protein level expression of SOX30	Age	1.067	1.009-1.130	**0.024**
	Gender	2.824	0.691-11.539	0.148
	Clinical stage	2.110	0.897-4.960	0.087
	Histological grade	0.935	0.496-1.762	0.836
	Tumor size	0.966	0.746-1.252	0.794
	Lymph node status	1.005	0.681-1.484	0.978
	SOX30 expression	1.283	1.004-1,639	**0.046**

Abbreviations: HR, hazard ratio; CI, confidence interval.

Supplementary Figure

Figure S1

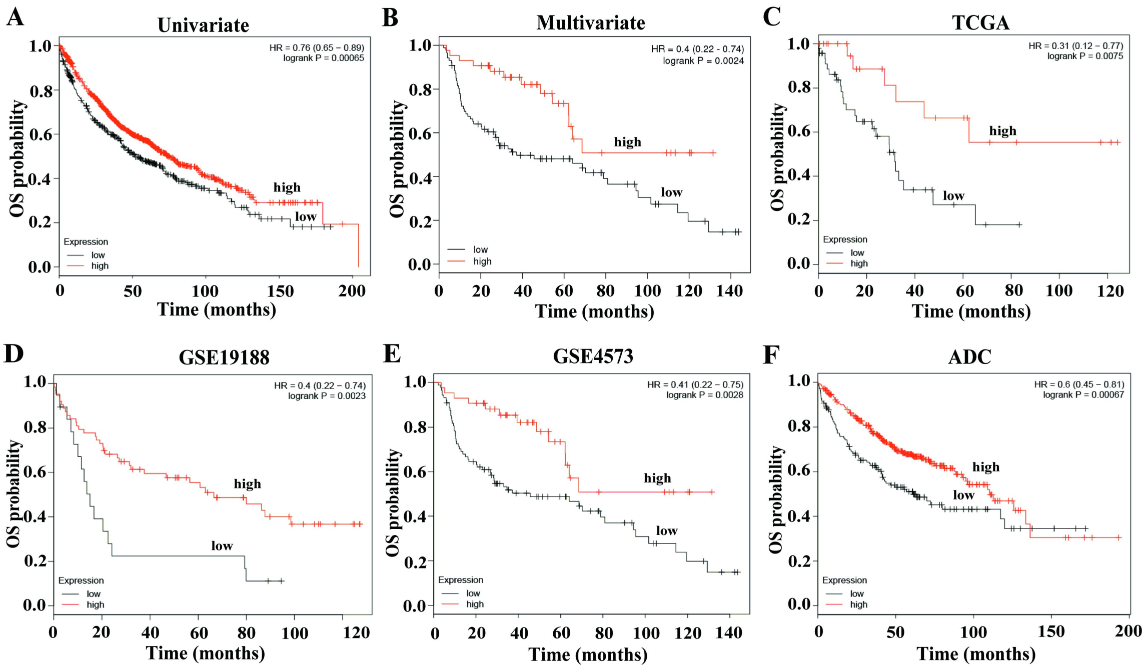

Supplementary Figure legends

Figure S1 High expression of SOX30 is correlated with better OS in lung cancer patients**.** (A) Kaplan-Meier survival analysis of SOX30 expression and overall survival (OS) in 1,432 lung cancer patients with Kaplan-Meier plotter (http: //kmplot.com/analysis/index). Release of the database was 2013 version; Auto select best cut-off was chosen in the analysis. (B) Cox-regression analysis of SOX30 expression in 130 lung cancer patients. Auto select best cut-off was chosen in the analysis. (C) Kaplan‑Meier survival analysis of SOX30 expression in 74 lung cancer patients of TCGA dataset. Auto select best cut-off was chosen in the analysis. (D) Kaplan-Meier survival analysis of SOX30 expression in 82 lung cancer patients of GSE19188 dataset. Auto select best cut-off was chosen in the analysis. (E) Survival analysis of SOX30 expression in 131 lung cancer patients of GSE4573 dataset. Auto select best cut-off was chosen in the analysis. (F) Kaplan‑Meier survival analysis of SOX30 expression in 487 lung ADC patients. Auto select best cut-off was chosen in the analysis.
